# Disrupted glymphatic function and its relationship with sleep and cognitive impairment in ME/CFS assessed via DTI-ALPS

**DOI:** 10.3389/fnins.2026.1875420

**Published:** 2026-06-19

**Authors:** Kiran Thapaliya, Sonya Marshall-Gradisnik, Maira Inderyas, Leighton Barnden

**Affiliations:** National Centre for Neuroimmunology and Emerging Diseases, Griffith University, Gold Coast, QLD, Australia

**Keywords:** diffusion weighted imaging, DTI-ALPS, glymphatic function, MRI, ME/CFS, sleep disturbance, cognitive dysfunction

## Abstract

The glymphatic system is a recently discovered brain waste clearance system that is mostly active during sleep and disengaged during wakefulness. Impaired glymphatic function leads to the deposition of metabolic waste products in the brain potentially causing inflammation leading to various symptoms in ME/CFS. While the glymphatic function has been assessed in other neurodegenerative diseases using ‘diffusion tensor imaging along the perivascular space’ (DTI-ALPS), it has not been studied in Myalgic Encephalomyelitis/Chronic Fatigue Syndrome (ME/CFS). This preliminary study investigates glymphatic function in 58 participants (ME/CFS = 31 and healthy controls = 27) using the DTI-ALPS index derived from DTI data acquired with 3 T MRI. The bilateral hemispheric DTI-ALPS index was estimated to assess glymphatic function, and an asymmetry index was calculated to determine interhemispheric asymmetry in glymphatic function. We found that the global DTI-ALPS index was significantly lower in ME/CFS patients compared to healthy controls (ME/CFS: 1.44 ± 0.086; healthy controls: 1.51 ± 0.11, *p* = 0.014), indicating reduced glymphatic function in ME/CFS. Examining the hemispheres separately, showed the right hemisphere DTI-ALPS index was lower in ME/CFS than healthy controls (ME/CFS = 1.41 ± 0.097; healthy controls = 1.49 ± 0.12; *p* = 0.009) but not different on the left. Additionally, we did not find any significant difference in asymmetry index between ME/CFS and healthy controls. We observed an association between the global DTI-ALPS index and severity of ‘sleep disturbance’ (*p* = 0.013, *r* = −0.47) and “impaired concentration” (*p* = 0.026, *r* = −0.43). This study demonstrated impaired glymphatic function in ME/CFS which may lead to symptoms such as cognitive dysfunction and sleep disturbance experienced by ME/CFS.

## Introduction

Myalgic Encephalomyelitis/Chronic Fatigue Syndrome (ME/CFS) is a condition which affects multiple body systems (Carruthers et al., 2003). The most common symptoms are cognitive impairment, fatigue, and sleep disturbance (Carruthers et al., 2011). These symptoms adversely affect the quality of life and social activities of individuals with ME/CFS. Emerging evidence suggests that an impaired waste clearance system in the brain may be a key contributor to neurological symptoms.

The glymphatic system is a recently discovered waste clearance system in the brain facilitated by the exchange of cerebrospinal fluid (CSF) and interstitial fluid (ISF), primarily through aquaporin-4 (AQP4) channels on astrocytic end feet (Wu et al., 2024; Huang et al., 2024). As CSF enters the brain interstitial space, it washes away metabolic waste like amyloid-*β* and distributes essential nutrients such as glucose, lipids, amino acids, and neuromodulators (Jessen et al., 2015; Iliff et al., 2012). Importantly, glymphatic function is strongly modulated by sleep (Gędek et al., 2023). Recent studies demonstrate that the glymphatic system becomes more active during sleep to enhance the clearance of metabolic waste (Gędek et al., 2023; Xie et al., 2013). Sleep disturbance/inadequate sleep impairs glymphatic function, leading to the deposition of metabolic waste products in the brain, causing cognitive dysfunction (Kamagata et al., 2022) and neuroinflammation (Jucker and Walker, 2013). Waste deposition, including amyloid-*β* and hyperphosphorylated tau, has been found in various neurodegenerative diseases, including Alzheimer’s disease (Rasmussen et al., 2022).

Glymphatic function has been evaluated using magnetic resonance imaging (MRI) techniques that visualise central nervous system fluid flow with gadolinium-based contrast agents (Lee et al., 2022). However, this method requires an invasive contrast injection, limiting its feasibility for routine clinical use. To address this limitation, a novel, non-invasive MRI-based method called “diffusion tensor imaging along the perivascular space” (DTI-ALPS) has been introduced (Taoka et al., 2017). The DTI-ALPS index quantifies glymphatic function by calculating the ratio of water diffusion parallel to the perivascular space versus diffusion perpendicular to it (Taoka et al., 2017). A higher DTI-ALPS index is interpreted as reflecting more efficient glymphatic clearance or function (Taoka et al., 2017). This technique offers several key advantages: it is non-invasive and requires no contrast agents, and can be acquired using standard clinical MRI scanners (Wood et al., 2024). DTI-ALPS demonstrates good test–retest stability, shows high intra-observer consistency (Siow et al., 2022) and is well-suited for broader adoption in both research and clinical practice (Taoka et al., 2022).

The DTI-ALPS index method has been used to assess the glymphatic function in neurodegenerative diseases like Alzheimer’s and multiple sclerosis, and other diseases such as cancer, Parkinson’s, type 2 diabetes mellitus, haemorrhagic stroke, and pain (Taoka et al., 2017; Yang et al., 2020; Bayoumi et al., 2024; Zhang et al., 2022; Wang et al., 2022). Importantly, the DTI-ALPS index is also associated with the Mini-Mental State exam, Montreal cognitive assessment, and motor control in Alzheimer’s, Parkinson’s disease, and older adults (Park et al., 2023; Bae et al., 2023). Moreover, the DTI-ALPS index was negatively correlated with Pittsburgh Sleep Quality Index (PSQI) scores, indicating that poorer sleep quality is associated with worse functioning of the glymphatic system (Ma et al., 2025). Disrupted sleep, such as difficulty in initiating sleep and insufficient sleep duration, directly impacts the glymphatic function (Ma et al., 2025; Sangalli and Boggero, 2023).

Although the glymphatic function has been studied in other neurodegenerative diseases, it has not yet been investigated in ME/CFS, despite the fact that over 90% of ME/CFS experiences poor sleep quality and cognitive dysfunction (Jackson and Bruck, 2012; Aoun Sebaiti et al., 2022). A recent prospective article proposed that dysregulation of the glymphatic system may contribute to the complex symptomatology observed in ME/CFS (Nemat-Gorgani et al., 2025). Impaired immune cells and elevated inflammatory mediators have been reported in ME/CFS (Komaroff and Lipkin, 2023). These effects within the perivascular space, potentially impair glymphatic clearance and thereby reduce the removal of inflammatory mediators, which may exacerbate neuroinflammation (Chen et al., 2025; Zou et al., 2024). Poor sleep and core cognitive impairment symptoms of ME/CFS are closely associated with glymphatic function in other conditions, yet this relationship has never been explored in ME/CFS, representing a critical gap in understanding its pathophysiology. Furthermore, although structural interhemispheric differences have been reported in ME/CFS (Zeineh et al., 2014; Thapaliya et al., 2022; Thapaliya et al., 2025) that could affect glymphatic dynamics, no study has investigated hemispheric asymmetry in glymphatic dysfunction in ME/CFS.

This preliminary study aims to investigate glymphatic function between individuals with ME/CFS compared to healthy controls using the DTI-ALPS index. In addition, we will examine asymmetry in glymphatic function in ME/CFS. Finally, this study will explore the association between glymphatic function and symptom severity in ME/CFS, providing novel insights into its underlying pathophysiology.

## Methods and materials

### Participant recruitment

The study was approved by the Griffith University Human Research Ethics Committee (Ref: 2022/666) and conducted in accordance with the principles outlined in the Declaration of Helsinki. Written informed consent was obtained from all participants prior to inclusion in the study.

This cross-sectional study was conducted at the National Centre for Neuroimmunology and Emerging Diseases (NCNED) on the Gold Coast, Queensland, Australia. Participants were recruited as previously described in Thapaliya et al. (2025). Individuals with ME/CFS were recruited if they met either the Canadian Consensus Criteria (CCC) or the International Consensus Criteria (ICC) for diagnosis (Carruthers et al., 2003; Carruthers et al., 2011), had received a formal ME/CFS diagnosis from a physician, and reported no prior history of COVID-19 infection. Healthy controls were individuals with no history of chronic illness or current medical conditions. All participants were aged between 18 and 65 years of age. A detailed medical history was obtained to exclude individuals with comorbidities, including mental illness, malignancies, autoimmune disorders, neurological diseases, or cardiovascular conditions. Female participants who were pregnant or breastfeeding were excluded. A total of 32 individuals with ME/CFS and 29 healthy controls were enrolled.

Symptom severity was assessed using a validated instrument. “Illness Duration”, “Pain”, and “Physical function” were extracted from SF36v2 (Alonso et al., 1995). The severity of ‘Pain’ and ‘Physical function’ was assessed on a 0 to 100-point scale, 0 being very severe and 100 being no symptom. We also extracted a ‘cognitive impairment’ score from the WHODAS [WHO Disability Assessment Schedule] version 2.0 (Üstün, 2012) scored on a 0–100-point scale, with higher scores indicating greater impairment (0 = no symptom, 100 = very severe). Additional measures included “Dr Bell’s Chronic Fatigue and Immune Dysfunction Syndrome (CFIDS) Disability Scale” (Bell, 1995), and “Modified Fatigue Impact Scale” (Guidelines MSCP, 1998) which provided an assessment of functional disability and fatigue impact. The severity ratings for “fatigue”, “impaired concentration”, and ‘sleep disturbance’ were obtained from the NCNED Research Registry questionnaire using a 0–5 ordinal scale (0 = none, 5 very severe) (Jessen et al., 2015). Detailed participant characteristics and corresponding clinical and symptom severity scores are provided in Tables 1, 2.

**Table 1:** Demographic and clinical characteristics of individuals with ME/CFS and healthy controls.

Demographic and clinical characteristics	ME/CFS (*n* = 31)	Healthy controls (*n* = 27)	*p*-value
Age	42.95 ± 13.51	37.92 ± 10.9	0.12
Sex (F/M)	21/10	19/8	N/A
BMI	24.43 ± 4.35	24.26 ± 2.56	0.96
Duration (years)	14.32 ± 11.29	N/A	N/A
Pain	51.07 ± 25.09	88.07 ± 18.05	<0.001
Cognitive	49.9 ± 16.93	5.53 ± 12.42	<0.001
Physical function	38.57 ± 27.51	93.65.0 ± 20.02	<0.001
Bell disability score	39.13 ± 14.43	97.91 ± 6.58	<0.001
MFIS	64.37 ± 10.76	7.48 ± 12.24	<0.001

F, Female; M, male; MFIS, Modified Fatigue Impact Scale.

**Table 2:** Prevalence of ME/CFS symptom severity scores obtained from a self-reported questionnaire.

Severity	None	Very mild	Mild	Moderate	Severe	Very severe	Missing data
Fatigue			3.4% (1/29)	41.4% (12/29)	34.4% (10/19)	20.7% (6/29)	2
Concentration		3.4% (1/29)	6.9% (2/29)	44.8% (13/29)	27.6% (8/29)	17.2% (5/29)	2
Sleep disturbance			20.7% (6/29)	41.4% 12/29	31% 9/29	6.9% (2/29)	2

Data of two ME/CFS were missing for each symptom severity measure.

## DTI data acquisition

Diffusion data was acquired using a 3 T Prisma MRI scanner (Siemens Healthcare, Erlangen, Germany) with a 64-channel head–neck coil (Nova Medical, Wilmington, USA). DTI data were obtained using 2-shell acquisition protocols: 30 directions at *b* = 1,000 s/mm^2^ and 66 directions at b = 2,500 s/mm^2^, along with 9 b = 0 scans. Other additional acquisition parameters included repetition time/echo time = 4100/75 ms, field of view (FOV) = 244 × 244, matrix = 122 × 122, voxel dimension 2.0 mm^3^, and 66 slices. For the estimation of DTI parameters, only the data acquired at b = 1,000 s/mm^2^ were used because this shell is optimal for standard DTI modelling.

### DTI data processing

DTI data was denoised using the “dwidenoise” command from MRtrix3,^1^ which estimates spatially varying noise levels using an algorithm based on random matrix theory (Cordero-Grande et al., 2019). Eddy current induced distortions and subject motion between and within DTI volumes were corrected using the “dwipreproc” command in MRtrix3, which performs affine registration of each DTI volume to a reference b = 0 image. Following the preprocessing, diffusion tensor modelling was performed using the ‘dtifit’ tool to compute the diffusion tensor and fractional anisotropy (FA) for each participant using the FMRIB Diffusion Toolbox (FDT), part of the FMRIB Software Library (FSL) (Smith et al., 2004).

### DTI-ALPS index estimation

**Figure 1:**
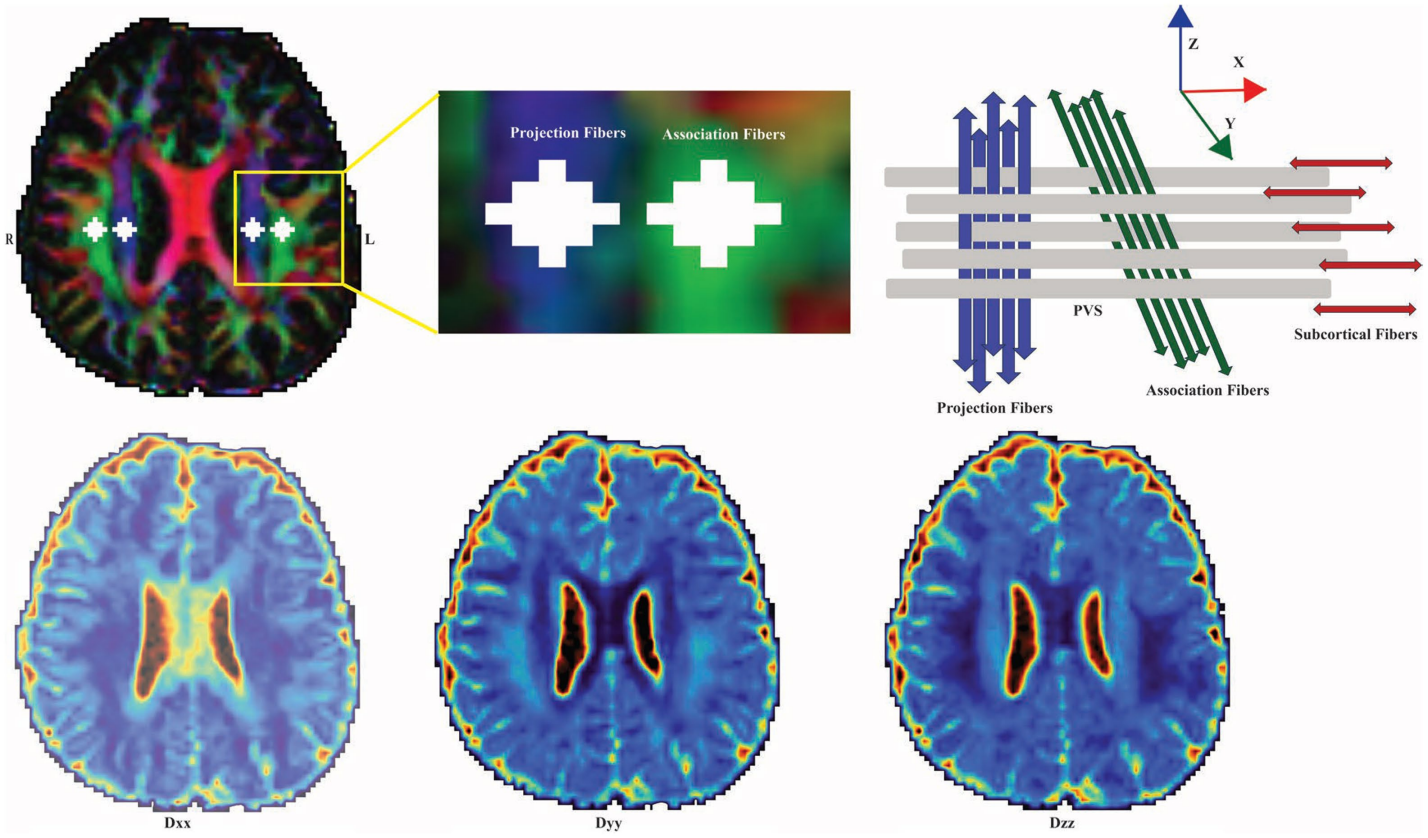
Position of regions of interest for DTI-ALPS index calculations on a color-coded fractional anisotropy map. Spherical ROIs (3 mm diameter) were positioned in the projection and association tracts. Dxx: left to right direction, Dyy: anterior to posterior direction, and Dzz: craniocaudal direction. PVS -perivascular space, L = left and R = right.

The procedure of DTI-ALPS index estimation is shown in Figure 1. Color-coded FA maps were used to precisely locate the DTI-ALPS regions of interest (ROIs). For each participant, ROIs were manually defined as a 3 mm diameter sphere using FSLeyes (see Figure 1). ROI delineation reliability was assessed via intraclass correlation coefficient (ICC) between two independent manual delineations; all ICC values exceeded 0.90, indicating excellent reproducibility. Within these ROIs, diffusivity along the x-axis was estimated as the mean tensor values in areas where perivascular spaces overlap with projection fibers (*D_xxproj_*) or association fibers (*D_xxassoc_*). These values can be extracted from the pre-processed DTI data. To standardise the index, the tensor value of projection fibers along the y-axis (*D_yyproj_*) (anterior to posterior) and the association fibers along the z-axis (*D_zzassoc_*) (craniocaudal) was also extracted from each ROI. The DTI-ALPS index was estimated for each hemisphere and was computed using the formula (Taoka et al., 2017)

$$\mathrm{DTI}-\text{ALPS index}=\frac{\text{mean}\;(D_{\text{xxproj}},D_{\text{xxasso}})}{\text{mean}(D_{\text{yyproj}},D_{\text{zzasso}})}$$

Asymmetry index was estimated using the formula (Li et al., 2025)

$$\text{Assytmetry Index}=\frac{\text{left}\;\mathrm{DTI}\_\text{ALPS}-\text{right}\;\mathrm{DTI}\_\text{ALPS}}{(\text{left}\;\mathrm{DTI}\_\text{ALPS}+\text{right}\;\mathrm{DTI}\_\text{ALPS})/2}$$

Finally, the bilateral (left and right hemisphere) DTI-ALPS indices were averaged to obtain a single, whole-brain DTI-ALPS index per participant. We estimated the DTI-ALPS index from 31 ME/CFS and 27 healthy controls. One ME/CFS and two healthy controls were excluded from the study due to motion artefact.

### Statistical analysis

Clinical scores for individuals with ME/CFS and healthy controls are presented as mean ± standard deviation. Group comparisons of the DTI-ALPS index between ME/CFS and healthy controls, as well as between left and right hemispheres within each group, were assessed using the general linear model. The Shapiro–Wilk test was used to evaluate data normality, which indicated that the DTI-ALPS index was normally distributed (*p* > 0.05). In contrast, clinical and severity measures (Table 1) were non-normally distributed; therefore, group analyses of clinical measures were performed using Quade’s non-parametric ANCOVA. To evaluate the association between glymphatic function (DTI-ALPS index) and symptom severity, we computed Spearman’s rank correlation coefficients between the DTI-ALPS index and severity scores for fatigue, sleep disturbance, and concentration, given the non-normal distribution of the severity measures. Age and sex were included as covariates in all group comparisons and correlation analyses to control for their potential confounding effects. All statistical analyses were conducted using SPSS Statistics (version 29). Multiple comparison correction was applied using the False Discovery Rate (FDR) method (Benjamini and Hochberg, 1995) across three distinct families of tests: (1) DTI-ALPS values, (2) clinical measures, and (3) correlations with severity scores. Statistical significance was defined as defined as *p-_FDR_* <0.05.

## Results

### Clinical and symptom characteristics

Detailed clinical symptom severity scores of ME/CFS are provided in Tables 1, 2. The majority of participants in this study were female ME/CFS (21 out of 31), healthy controls (19 out of 27). The age and body mass index (BMI) were comparable between ME/CFS and healthy controls (see Table 1). Most of the participants completed an online questionnaire before visiting the MRI scanning sites. ME/CFS experienced significantly higher ‘pain’ (*p* = <0.001), and impaired ‘cognitive function’, ‘physical function’, ‘Bell disability’ score, and ‘Modified Fatigue Impact Scale’ (all *p* = <0.001) compared to healthy controls (see Table 1).

The most commonly reported symptoms among ME/CFS were ‘fatigue’ (28 out of 29, 96.6%); ‘impaired concentration’ (26 out of 29, 89.6%), and ‘sleep disturbance’ (23 out of 29, 79.3%). These symptoms were rated from moderate to very severe (see Table 2). Only 3 out of 29 (10.3%) ME/CFS reported impaired concentration of very mild or mild severity (see Table 2).

### DTI-ALPS index ME/CFS vs. healthy controls

**Figure 2:**
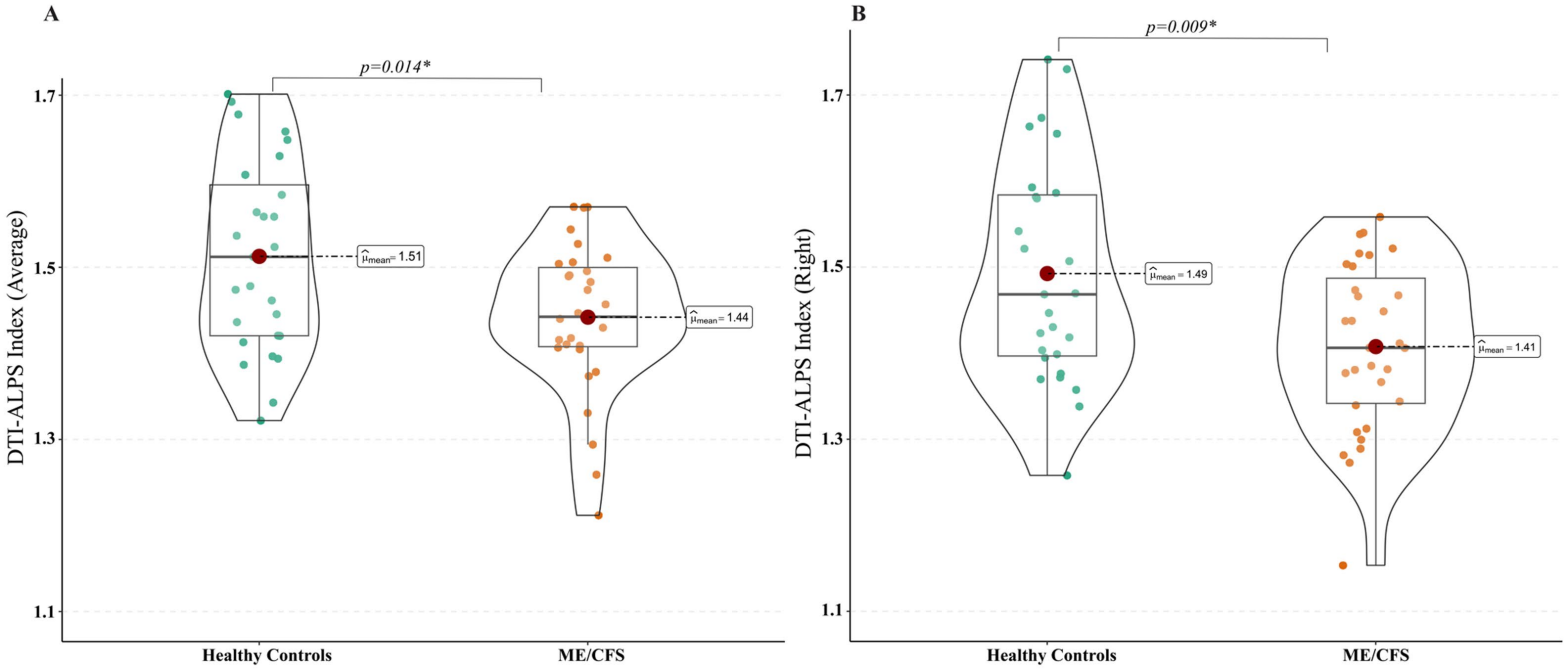
DTI-ALPS index estimated in ME/CFS and healthy controls. **(A)** Demonstrate significantly lower DTI-ALPS index in ME/CFS compared to healthy controls. **(B)** Shows significantly lower DTI-ALPS index in the right hemisphere of ME/CFS compared to healthy controls.

We found significantly lower global DTI-ALPS index in ME/CFS (1.44 ± 0.086) compared to healthy controls (1.51 ± 0.11, *p* = 0.014, *p-_FDR_* = 0.028) (see Figure 2A). When comparing unilateral DTI-ALPS index between ME/CFS and healthy controls, we found significant difference in the right DTI-ALPS index ME/CFS (1.41 ± 0.097) compared to healthy controls (1.49 ± 0.12; *p* = 0.009, *p-_FDR_* = 0.028) (see Figure 2B; Table 3). However, no significant difference was found in the left hemisphere DTI-ALPS index between the two groups (see Table 3).

**Table 3:** Statistical comparison of DTI-ALPS indices between ME/CFS and healthy controls.

Metrics	ME/CFS	Healthy controls	*p*-value	Confidence interval		Cohen’s *d* effect size
				Lower bound	Upper bound	
Group comparison						
Global DTI-ALPS	1.44 ± 0.86	1.51 ± 0.11	0.014*	−0.122	−0.014	0.107
Right DTI-ALPS	1.41 ± 0.97	1.49 ± 0.12	0.009*	−0.144	−0.022	0.119
Left DTI-ALPS	1.47 ± 0.11	1.53 ± 0.13	0.116	−0.119	0.013	0.045
Left vs. Right within ME/CFS						
Left DTI-ALPS	1.47 ± 0.11		0.012*	0.015	0.0121	0.66
Right DTI-ALPS	1.40 ± 0.097					
Left vs. Right within Healthy Controls						
Left DTI-ALPS	1.53 ± 0.13		0.261	−0.030	0.111	0.309
Right DTI-ALPS	1.49 ± 0.12					
Asymmetry index						
	ME/CFS	Healthy controls				
Asymmetry Index DTI-ALPS	0.047 ± 0.08	0.026 ± 0.088	0.357	−0.025	0.068	0.016

*represents the statistically significance after adjusting for multiple comparisons using the false discovery rate.

### Asymmetric DTI-ALPS index in ME/CFS and health controls

**Figure 3:**
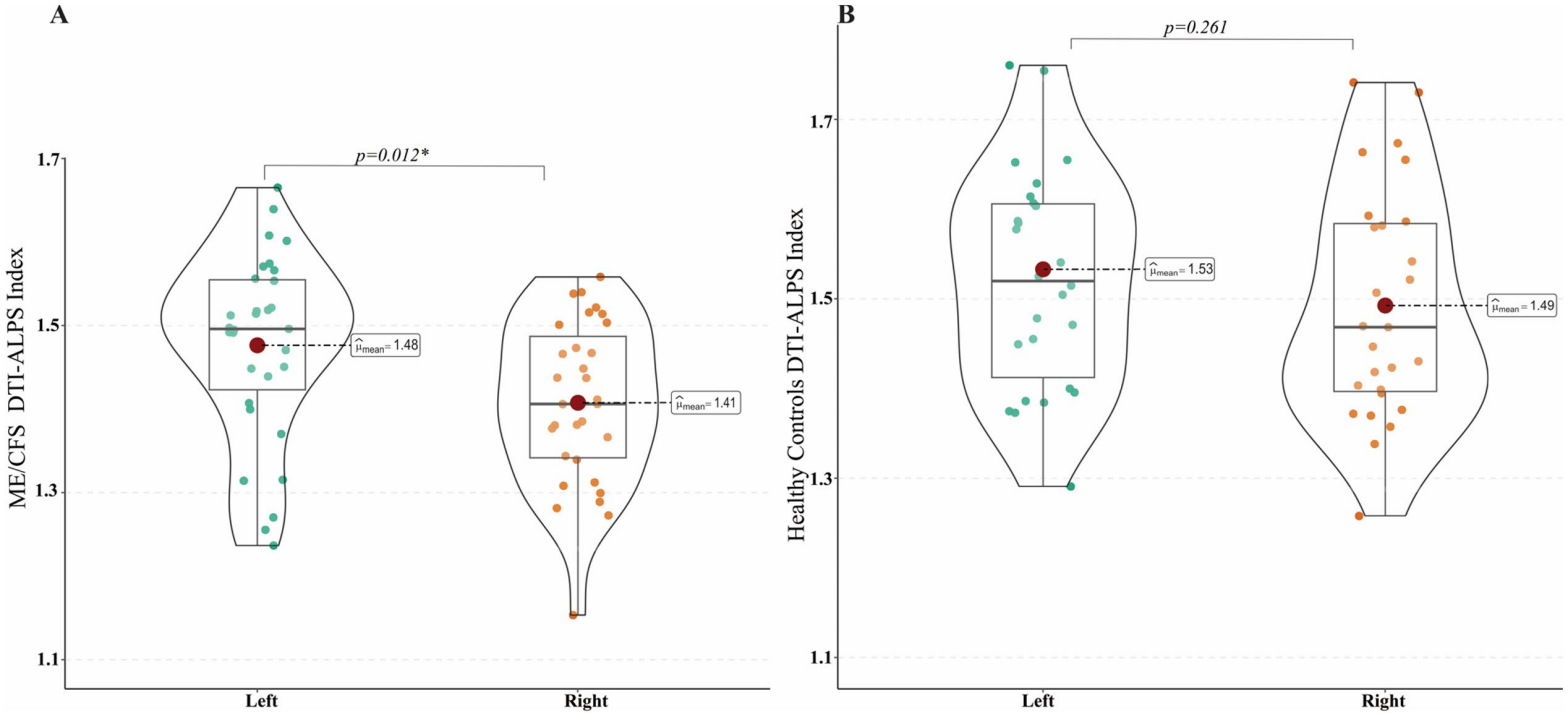
The DTI-ALPS index values in the left and right hemispheres. **(A)** Shows significantly lower DTI-ALPS index in the right hemisphere of ME/CFS compared to the left hemisphere. **(B)** Shows no significant differences in the DTI-ALPS index in healthy controls between the left and right hemispheres.

We found a significantly asymmetric DTI-ALPS index among ME/CFS (see Figure 3A). The right hemisphere DTI-ALPS index was significantly lower compared to the left hemisphere (left: 1.47 ± 0.11, right: 1.40 ± 0.097, *p* = 0.012, *p-_FDR_* = 0.028). In contrast, no significant difference in the DTI-ALPS index were observed between left and right hemisphere (left: 1.53 ± 0.13, right: 1.49 ± 0.12, *p* = 0.261) in healthy controls (see Figure 3B; Table 3). Additionally, we observed a trend toward rightward asymmetry, however, the asymmetry index was not significant between ME/CFS and healthy controls (ME/CFS = 0.047 ± 0.08; healthy controls = 0.026 ± 0.088; *p* = 0.357).

### Correlations with severity measures

**Figure 4:**
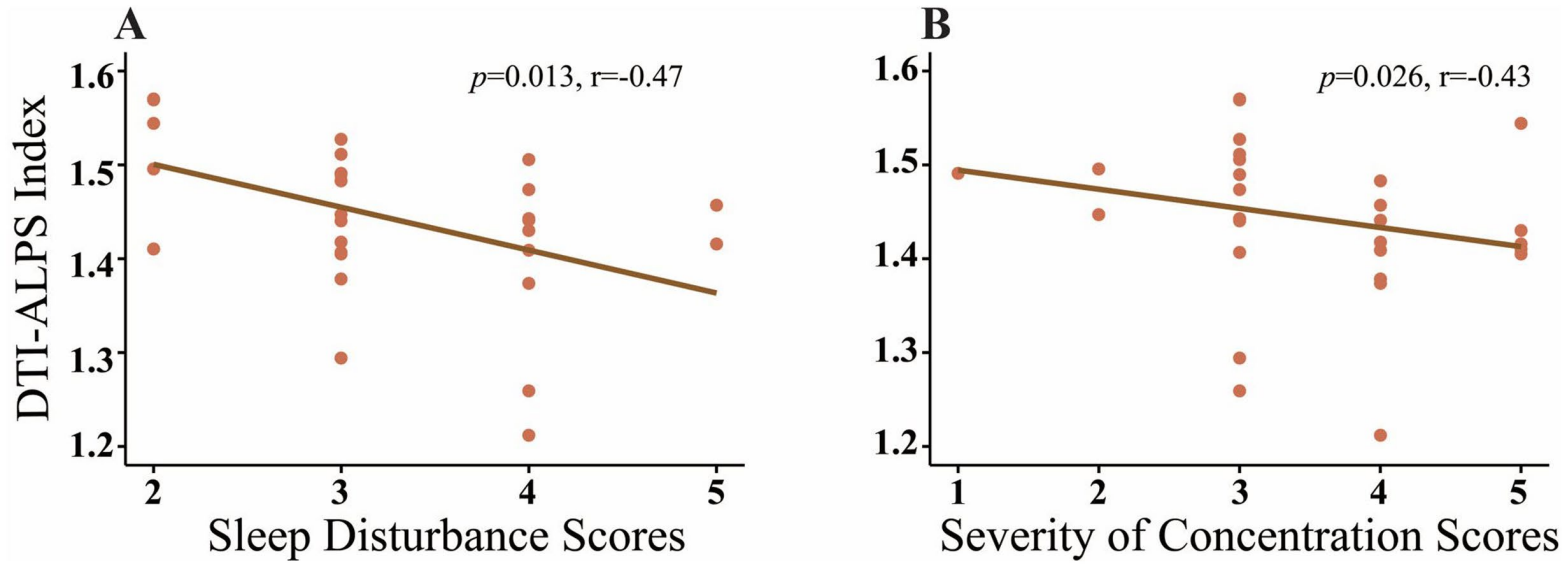
Demonstrate the correlation between DTI-ALPS index and severity measures. **(A)** Shows global DTI-ALPS index was significantly associated with sleep disturbance scores. **(B)** Demonstrate global DTI-ALPS index was significantly associated severity of concentration.

In ME/CFS we found a significant negative association between “sleep disturbance” severity and global DTI-ALPS index (*p* = 0.013, *r* = −0.47; see Figure 4A). Global DTI-ALPS index was also inversely correlated with ‘impaired concentration’ (*p* = 0.026, *r* = −0.43; see Figure 4B). However, there were no associations between the unilateral DTI-ALPS index or the global DTI-ALPS index and other symptom severity measures including, “fatigue”, “severity”, “illness duration”, “pain”, “cognitive”, and “physical function” in ME/CFS.

## Discussion

Our study is among the first to evaluate the DTI-ALPS index in ME/CFS, revealing a potential reduction in glymphatic function. We found that the DTI-ALPS index was significantly lower in ME/CFS compared to healthy controls, indicating impaired glymphatic clearance. Furthermore, a lower DTI-ALPS index was significantly associated with sleep disturbance and impaired concentration. These findings suggest that glymphatic dysfunction could potentially contribute to the neurological symptoms experienced by ME/CFS.

Reduced glymphatic function measured by using the DTI-ALPS method has been reported in brain-related diseases such as Parkinson’s disease, cerebral small vessel disease, Alzheimer’s, dementia, and multiple sclerosis (Taoka et al., 2017; Yang et al., 2020; Bayoumi et al., 2024; Zhang et al., 2022; Wang et al., 2022). However, due to the current lack of prior studies on glymphatic function in ME/CFS, direct comparison with our findings is not yet possible. The observed reduced glymphatic function in ME/CFS could be due to multiple factors, including sleep deprivation, reduced physical activity, elevated stress, Omega-3, or hypertension (Gędek et al., 2023). It is well established that glymphatic function is more active during sleep and plays a critical role in clearing metabolic waste from the brain (Jessen et al., 2015). Disrupted or insufficient sleep experienced by ME/CFS (Jackson and Bruck, 2012) may directly impair glymphatic clearance. Additionally, other common ME/CFS symptoms may further suppress glymphatic function. Kimura et al. (2023) reported that free water corrected DTI metrics were observed over a wider extent in ME/CFS. Since, free water is present in the deep brain parenchyma, this could also affect the glymphatic system in ME/CFS. Physical activity (Hayes et al., 2025), stress (Balinas et al., 2021), hypertension (Denu et al., 2025) that are common symptoms in ME/CFS have all been associated with reduced glymphatic function (Gędek et al., 2023). Animal studies in mice demonstrated that voluntary exercise enhances glymphatic clearance (von Holstein-Rathlou et al., 2018) while higher stress impairs glymphatic flow (Wei et al., 2019). Similarly, in humans, hypertension patients showed reduced perivascular space compared to healthy controls (Kikuta et al., 2022) resulting in glymphatic dysfunction. Furthermore, in ME/CFS, a lack of Omega-3 (Maes et al., 2005) may further disrupt glymphatic function. Omega-3 acids are known to improve glymphatic function by promoting waste removal and facilitating chemical exchange in the brain (Zhang et al., 2020).

Additionally, we found that only the right hemisphere DTI-ALPS index was significantly lower in ME/CFS compared to healthy controls, with no differences observed in the left hemisphere. This kind of hemispheric asymmetry in the DTI-ALPS index has been previously reported in patients with temporal lobe epilepsy, Parkinson’s disease, and in amyotrophic lateral sclerosis compared to healthy controls (Zhang et al., 2022; Li et al., 2025; Baek et al., 2025). ME/CFS is a progressive multisystem illness, and factors such as severe physical deconditioning and chronic sleep disturbances may contribute to this asymmetry. Furthermore, within ME/CFS, we found that right hemisphere DTI-ALPS was significantly lower compared to the left indicating lateralised impairment of glymphatic function. Similarly, in Parkinson’s disease, the right hemisphere DTI-ALPS index was lower than the left (Li et al., 2025). Supporting the plausibility of asymmetry in ME/CFS, prior neuroimaging studies have found an asymmetric cortical thickness and cortical volume (Thapaliya et al., 2022; Thapaliya et al., 2025).

This study further confirms the lateralised dysfunction of glymphatic function in ME/CFS. However, the underlying mechanism driving this right-hemisphere predominant glymphatic dysfunction in ME/CFS remains unknown and further research is needed.

### Correlations with severity measures

Our study found a significant negative correlation between ‘sleep disturbance’ scores and the DTI-ALPS index in ME/CFS, indicating that greater severity of sleep disruption is associated with reduced glymphatic function in ME/CFS. Prior research in Alzheimer’s, traumatic brain injury, stroke, migraine, and epilepsy has consistently shown that sleep problems and disorders disrupt glymphatic function (Christensen et al., 2021). Importantly, a recent study has demonstrated that glymphatic clearance is 90% more active during sleep than during wakefulness (Xie et al., 2013). Therefore, one of the core functions of sleep is to clear the brain’s metabolic waste via the glymphatic system that accumulates during wakefulness (Jessen et al., 2015). This strongly supports our findings in ME/CFS because sleep disturbance is one of the major symptoms of this illness. This could accumulate metabolic waste products in the brain, potentially triggering neuroinflammation and contributing to the diverse neurological symptoms experienced by ME/CFS. Furthermore, we found a significant negative correlation between ‘impaired concentration’ and DTI-ALPS index in ME/CFS, indicating that reduced glymphatic function is associated with greater cognitive impairment. This relationship has been observed in Alzheimer’s (Kamagata et al., 2022), Schizophrenia (Wu et al., 2025), and cerebral small vessel disease (Tang et al., 2022). In ME/CFS, one of the possible underlying mechanisms of cognitive impairment could be the accumulation of metabolic waste products in the brain due to glymphatic dysfunction. Studies have shown that the glymphatic system not only clears pathological proteins but also iron (Iliff et al., 2012; Ishida et al., 2022). Due to glymphatic dysfunction, deposition of these substances and inflammatory mediators may trigger and further increase neuroinflammation (Jucker and Walker, 2013). Therefore, glymphatic dysfunction contributes to the deposition of metabolic waste products and sustained neuroinflammation, potentially causing neurological symptoms in ME/CFS.

Despite these valuable findings, our study has some limitations. First, this is a cross-sectional study with a relatively small sample size. Second, a longitudinal study is needed to investigate whether glymphatic dysfunction is progressive or stable. Third, glymphatic function is influenced by handedness (Perlaki et al., 2025) and sleeping position (Lee et al., 2015), which were not controlled for or measured in this study. Furthermore, the estimation of DTI-ALPS index could also be affected by manual ROI placement and algorithm-dependent calculation, for example FA based versus fiber tractography, and by sex (Clark et al., 2024). However, due to the uneven sex distribution and small sample size of our cohort, we were unable to explore these dimensions. Therefore, future studies should prioritise a larger sample size with balanced cohorts to validate our findings.

## Conclusion

We provide the first evidence of glymphatic dysfunction in ME/CFS compared to healthy controls and its association with symptom severity measures, including sleep disturbance and impaired concentration. These findings suggest that glymphatic dysfunction may contribute to the accumulation of metabolic waste products in the brain that may trigger or sustain neuroinflammation, ultimately leading to the neurological symptoms experienced by ME/CFS.

## Data Availability

The raw data supporting the conclusions of this article will be made available by the authors, without undue reservation.

## Footnotes

1
https://www.mrtrix.org/
